# Grazing Is Associated with ADHD Symptoms, Substance Use, and Impulsivity in a Representative Sample of a Large Metropolitan Area in Brazil

**DOI:** 10.3390/nu15132987

**Published:** 2023-06-30

**Authors:** Andreea I. Heriseanu, Dean Spirou, Carlos E. F. Moraes, Phillipa Hay, Rosely Sichieri, Jose C. Appolinario

**Affiliations:** 1 eCentreClinic, School of Psychological Sciences, Macquarie University, Wallumattagal Campus, Macquarie Park, NSW 2109, Australia; 2 School of Medicine, Western Sydney University, Sydney, NSW 2214, Australia; d.spirou@westernsydney.edu.au (D.S.); p.hay@westernsydney.edu.au (P.H.); 3 Discipline of Clinical Psychology, Graduate School of Health, University of Technology Sydney, Sydney, NSW 2007, Australia; 4 Translational Health Research Institute, Western Sydney University, Penrith, NSW 2751, Australia; carloseduardofm09@gmail.com; 5 Obesity and Eating Disorders Group, Institute of Psychiatry, Federal University of Rio de Janeiro, Rio de Janeiro 22290-140, Brazil; jotappo@gmail.com; 6 Mental Health Service, South West Sydney Local Health District, Campbelltown, NSW 2560, Australia; 7 Social Medicine Institute, State University of Rio de Janeiro, Rio de Janeiro 22290-140, Brazil; rosely.sichieri@gmail.com

**Keywords:** grazing, middle-income country, epidemiology, eating behaviours, substance use, tobacco, alcohol, attention-deficit/hyperactivity disorder, ADHD, impulsivity

## Abstract

Grazing is a clinically relevant eating behaviour, especially when it presents with a sense of loss of control (compulsive grazing). There is evidence that other disordered eating patterns are associated with problematic substance use and impulsivity-related conditions, such as attention-deficit/hyperactivity disorder (ADHD). This overlap contributes to higher psychopathology and treatment complications. Less is known about grazing, and most information originates in high-income countries. Hence, we sought to investigate relationships between grazing, tobacco and alcohol use, ADHD, and impulsivity in a large representative sample from Brazil. Data were collected by trained interviewers from adults (N = 2297) through an in-person household survey based on a stratified and clustered probability sample. We found significant associations between compulsive grazing and problematic alcohol use (OR = 3.02, 95% CI: 1.65, 5.53), ADHD (OR = 8.94, 95% CI: 5.11, 15.63), and smoking (OR = 1.67, 95% CI: 1.12, 2.47), with impulsivity contributing to the first two relationships. The substantial association with ADHD suggests that other executive functions may promote disordered eating, possibly expressed through difficulties in adhering to regular meals. Clinically, these findings highlight the importance of assessing problematic eating patterns, such as compulsive grazing, in those presenting with difficulties with substance use or impulsivity, and vice versa.

## 1. Introduction

Substance use disorders and disordered eating share neural and behavioural commonalities [1,2]. Examining the extent and limits of these commonalities and their mechanisms is key for understanding these overlapping conditions and improving assessment and treatment. A variety of overeating patterns can reflect “addictive-like” eating, including grazing [1]. Grazing is the unstructured, repetitive eating of small amounts of food over a long time period outside of planned meals and snacks and/or not in response to hunger or satiety sensations [3]. Grazing consists of two subtypes: compulsive and non-compulsive. Compulsive grazing occurs where there is a perceived inability to resist or stop grazing [4], and it is significantly less common than non-compulsive grazing (10.2% in the general Australian population [5] and in the general Brazilian population [6]). Compulsive grazing is associated with greater eating disorder psychopathology, increased distress, lower mental health-related quality of life, and reduced treatment success in patients with high body weight [5,7,8,9]. Non-compulsive grazing, referring to eating repetitively in a distracted fashion, is common in the population (depending on the definition used, rates range between 38% and 90% [5,10]) and is generally not associated with deleterious effects. Thus far, research on conditions co-occurring with grazing has focused on eating disorders and high body weight and has been predominantly conducted in high-income countries. As such, there are important knowledge gaps pertaining to relationships between grazing and high-burden conditions other than eating and weight disorders, especially in the context of global health epidemiology.

Alcohol and tobacco are two of the most commonly used substances worldwide [11]. The harmful use of alcohol is a major cause of disability and death, bringing significant social and economic losses to individuals and society at large [12]. There is a high prevalence of alcohol consumption and heavy episodic drinking in low- and middle-income countries, such as Brazil [13,14,15]. Tobacco use is a major risk factor for cardiovascular and respiratory diseases, cancer, and many other serious health conditions; most tobacco-related deaths occur in low- and middle-income countries [16], and the rate of decline has slowed in recent years [17]. Both alcohol and tobacco use are common in attention-deficit/hyperactivity disorder (ADHD); those with ADHD are more likely to develop problematic alcohol and/or tobacco use [18], while ADHD is a common co-morbidity in people with substance use disorders [19].

Both tobacco and alcohol use are associated with impulsivity [20,21]. Impulsivity encompasses behaviours or responses that are poorly conceived, premature, and inappropriate and that frequently result in undesirable outcomes [22], and it is a transdiagnostic feature of substance use disorders, ADHD, and other psychiatric conditions [23]. Several eating disorders, such as bulimia nervosa and binge eating disorder, are also associated with impulsivity [24]. Both food-related impulsivity [25] and general impulsivity [26] occur in objective binge eating (i.e., involving an objectively large amount of food and a sense of loss of control over eating) and complicate its treatment [27]. When impulsivity is present in ADHD, it is associated with overeating [28]. Additionally, tobacco use and binge drinking, as well as alcohol use disorder, are higher in persons who binge eat [29,30,31]. Research suggests that the overlap of disordered eating and substance use is associated with more severe eating disorder symptoms and general psychopathology, poor treatment outcomes, and high relapse rates [32] and that it may form part of a pattern of impulsive behaviour. Studies have reported co-morbidity between ADHD, substance use disorders, and binge-type eating disorders, including studies in Brazil [33]. Comorbid eating disorders and ADHD increase the risk for a substance use disorder [34], and ADHD is considered an important mediator or aetiological factor in the relationship between eating disorders and substance use [32]. These findings suggest multiple relationships between problematic food and substance consumption and neuropsychiatric conditions, potentially underpinned by impulsivity.

As highlighted above, research into problematic substance use, impulsivity, and food intake has predominantly focused on objective binge eating, with less research attention directed towards compulsive grazing and other problematic atypical eating behaviours which also exhibit impulsive/compulsive dimensions, such as subjective binge eating (presenting with a sense of loss of control without an objectively large amount of food consumed) [35] and food addiction (an escalation in the intake of and pronounced cravings for processed palatable foods) [36].

The repetitive consumption pattern that characterises grazing bears similarities to behaviours encountered in substance use disorders; for example, “steady” drinking, where individuals consume alcohol continuously throughout the day, has been identified as a clinically relevant alcohol use pattern associated with a severe alcohol use history [37]. Likewise, chain-smoking (i.e., smoking one cigarette right after another) is common in those who use tobacco, and it is associated with higher impulsivity [38]. Given its unplanned, repetitive nature and its occurrence outside of hunger/satiety signals, grazing (especially when incorporated with a sense of loss of control) may also be conceptually regarded as a behaviour performed automatically in response to internal emotional states and/or food-related cues in the environment [39] potentially resulting from failures of self-regulation. 

In summary, problematic relationships exist between disordered eating, ADHD symptoms, and substance use, potentially implicating impulsivity. However, despite robust theoretical links, the relationship between grazing, tobacco and alcohol use, and impulsivity has not been previously examined. Additionally, information on grazing originating from middle-income countries is scarce. Therefore, the aims of the current study were (1) to identify the relationships between compulsive and non-compulsive grazing and self-reported ADHD symptoms, common licit substance use (alcohol and tobacco), and impulsivity and (2) to examine whether impulsivity is a contributor to relationships between grazing, ADHD symptoms, and substance use in a large representative sample sourced from a Brazilian metropolitan city.

## 2. Materials and Methods

### 2.1. Participants

This study was part of a larger survey of binge eating and related variables in the general population of the city of Rio de Janeiro, Brazil, a large city in a middle-income country [33]. The study was an in-person household survey. The survey was based on a stratified and clustered probability sample selected in three stages: census enumeration areas, households, and eligible adults. Adults aged 18–60 were included, and pregnant and breastfeeding women were excluded. The study was approved by the research ethics committee of the Institute of Psychiatry, Federal University of Rio de Janeiro. All study participants provided written informed consent.

### 2.2. Procedure

Following sample selection, each selected household was contacted by an interviewer and invited to participate in a survey about eating behaviour and mental health. Data were collected from September 2019 to February 2020. Of 2985 eligible households, 688 declined to participate. The participation rate was 77%, with a total of 2297 individuals participating. Trained interviewers invited participants to complete a research questionnaire on a tablet and measured their height and weight.

### 2.3. Measures

*Demographics.* The following characteristics were collected: self-defined gender, age, self-defined race/ethnicity, marital status, number of children, education, employment status, and income. 

*Anthropometry.* Weight was measured on a digital scale (Plenna^®^, São Paulo, Brazil), and height was measured using a portable stadiometer (model 206; Seca^®^, Hamburg, Germany). Participants were weighed and measured barefoot, wearing light clothing, with their arms hanging alongside the body. Body mass index (BMI) was calculated as weight (kg)/height^2^ (m^2^) and was categorised according to the WHO cut-offs (underweight: <18.5; healthy weight: 18.5–24.9; overweight: 25–29.9; obesity: ≥30) [40].

*Grazing.* The Short Inventory of Grazing (SIG; [8]) is a validated two-item measure of grazing frequency that measures the presence and frequency of grazing in general and “compulsive” grazing, which is characterised by a sense of loss of control. Frequency is rated on a seven-point scale ranging from “none at all” to “eight or more times per week”. Mutually exclusive categories were created on the basis of the SIG: regular non-compulsive grazing (grazing without loss of control, occurring at least once a week), regular compulsive grazing (grazing with loss of control, occurring at least once a week), and no grazing ( grazing less than once a week); these categories were similar to those used in previous epidemiological surveys [5,6]. The SIG has sound psychometric properties and has been cross-culturally adapted to the Brazilian context, and it has been validated in a Brazilian sample [41].

*ADHD symptoms.* To assess symptoms of ADHD, the Brazilian version of the World Health Organization Adult Attention-Deficit/Hyperactivity Disorder Self-Report Screening Scale (ASRS) for the DSM-5 was used [42]. Probable ADHD was defined as an ASRS score of≥14.

*Alcohol use.* Alcohol use problems were screened using the Alcohol Use Disorders Identification Test (AUDIT), Brazilian version [43]. Problematic alcohol use was defined as an AUDIT score of >8.

*Tobacco use.* Tobacco use was assessed by asking three questions from the Brazilian National Health Survey [44]: cigarette smoking status (current, past, or never smoked), the number of cigarettes smoked per day (if current), and how long ago participants stopped smoking (if past smoking was reported). Lifetime smoking was marked as present if participants reported either current or past smoking.

*Impulsivity.* This was assessed with a single question that was previously used in an epidemiological study of this trait in the general population: “Most of the time throughout your life, regardless of the situation or whom you were with, have you often done things impulsively?” This question was validated and showed good psychometric properties for the evaluation of impulsivity [23].

### 2.4. Statistical Analysis

Data were weighted to adjust for differences in the probability of selection and for nonresponse, considering the probability of selection in each of the sampling stages, including within-household selection. To account for the effect of the complex design of the survey, the complex samples procedure was utilised in SPSS v28.0. Descriptive statistics (mean (M), standard error (SE), and frequencies) were obtained with 95% confidence intervals (CIs) for demographic variables, and the crosstabs procedure was used to generate % across categories. A series of binary logistic regressions was used to assess associations as odds ratios (OR) between grazing and the variables of interest (alcohol and tobacco use, impulsivity, and positive ADHD screening). These were conducted with and without potential covariates (gender, age, race, marital status, income, education, BMI, and the presence of an eating disorder). The influence of impulsivity on the observed relationships between grazing and alcohol and tobacco use and positive ADHD screening was tested through multiple logistic regression. Alpha was set at 0.05. Only 0.3% of cases were missing; hence, no imputation procedure was applied. 

## 3. Results

### 3.1. Sociodemographic and Clinical Variables

Approximately 52% of the participants identified as women, the mean participant age was 38.18 years (SE = 0.44), and the mean BMI was 27.61 kg/m^2^ (SE = 0.19). Most participants described themselves as “mixed race/ethnicity” (43.2%). The majority were married (54.2%), in full-time employment (63.5%), and had completed high school-equivalent education (46.3%). The median monthly income was BRL 1001–3000 (equivalent to USD 190–568), consistent with the median income distribution in the general population of Brazil. Overall, 10.2% reported regular compulsive grazing, whereas 29.8% reported regular non-compulsive grazing. Almost half (42.9%) reported impulsivity, approximately 25% reported lifetime smoking, 8.1% were categorised as having problematic alcohol use, and 4.5% had a positive ADHD screening, consistent with other studies using screening instruments in a Brazilian sample [45] and with global prevalence estimates [46]. For more details, see Table 1. Frequencies reflecting the overlap between the different grazing subtypes and problematic alcohol use, lifetime smoking, impulsivity, and positive ADHD screening are shown in Table 2. 

### 3.2. Impulsivity

Impulsivity was significantly associated with grazing (*p* < 0.001). Those who reported impulsivity had 2.75 times the odds (95% CI: 1.89, 4.00) of engaging in regular compulsive grazing rather than no grazing compared with those who did not report this item, whereas no significant difference in odds was found between those with non-compulsive grazing and those with no regular grazing (OR = 1.15, 95% CI: 0.88, 1.51). This did not significantly change when controlling for potential covariates (see Table 3).

### 3.3. ADHD Screening

**Figure 1:**
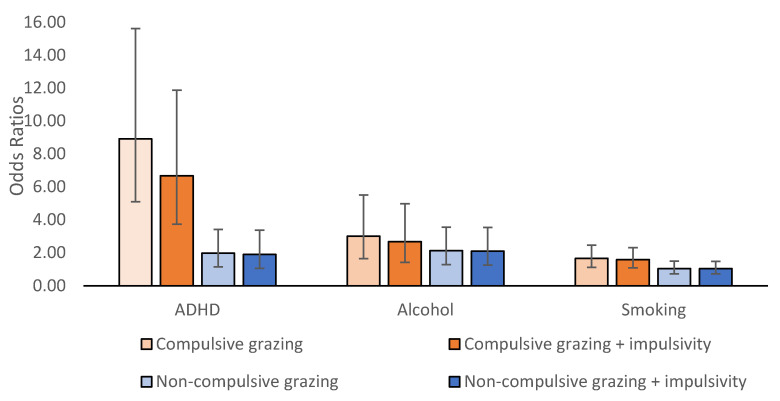
Associations between ADHD symptoms, alcohol use, smoking, and grazing and the effect of impulsivity on these associations. Note: no regular grazing was used as the reference category.

Those classified as probable ADHD cases had 8.94 times the odds (95% CI: 5.11, 15.63, *p* < 0.001) of engaging in compulsive grazing than no grazing and 1.98 times the odds (95% CI: 1.15, 3.43, *p* < 0.001) of engaging in non-compulsive grazing than no grazing compared with non-ADHD cases. No significant differences were observed when these analyses were repeated with the inclusion of potential covariates (Table 3) except for a decrease in the strength of the association between positive ADHD screening and compulsive grazing, which was likely due to adjusting for BMI. Positive ADHD screening was also significantly associated with impulsivity (OR = 6.86, 95% CI: 3.63, 12.95, *p* < 0.001), and appeared to partially account for the relationship between probable ADHD and compulsive grazing (controlling for impulsivity, compulsive grazing OR = 6.69, 95% CI: 3.76, 11.89, *p* < 0.001, whereas the relationship with non-compulsive grazing was only minimally reduced, OR = 1.90, 95% CI: 1.07, 3.38, *p* < 0.001); see Figure 1. The association between positive ADHD screening and alcohol use was similar to that between positive ADHD screening and grazing, such that those classified as probable ADHD cases had 5.12 times the odds (95% CI: 3.11, 8.44, *p* < 0.001) of having problematic alcohol use. The association with lifetime smoking was not significant (OR = 1.40, 95% CI = 0.89, 2.21, *p* = 0.146).

### 3.4. Alcohol Use

A significant association existed between problematic alcohol use and grazing (*p* = 0.002). Specifically, participants with problematic alcohol use had 3.02 times the odds (95% CI: 1.65, 5.53) of engaging in compulsive grazing and 2.14 times the odds (95% CI: 1.29, 3.56) of engaging in non-compulsive grazing than no grazing, respectively, compared with those without problematic alcohol use. No significant differences were observed when these analyses were repeated with the inclusion of potential covariates (Table 3). Impulsivity was also a significant predictor of problematic alcohol use (OR = 1.82, 95% CI: 1.32, 2.46, *p* < 0.001) and appeared to partially account for the relationship between compulsive grazing and problematic drinking (controlling for impulsivity, compulsive grazing OR = 2.68, 95% CI: 1.43, 4.99, *p* < 0.001, whereas the relationship with non-compulsive grazing was only minimally reduced, OR = 2.11, 95% CI: 1.26, 3.55, *p* < 0.001); see Figure 1.

### 3.5. Smoking

In terms of cigarette smoking, a significant association also existed between current or past smoking and grazing (*p* = 0.010). Participants with lifetime smoking had 1.67 times the odds (95% CI: 1.12, 2.47) of engaging in compulsive grazing than no grazing compared with those without lifetime smoking. No significant difference in odds was found between those with and without lifetime smoking in terms of non-compulsive grazing (OR = 1.05, 95% CI: 0.73, 1.50). No significant differences were observed when these analyses were repeated with the inclusion of potential covariates (Table 3). Impulsivity was not significantly associated with lifetime smoking (OR = 1.27, 95% CI: 0.96, 1.69, *p* = 0.099) and did not appear to be influential in the relationship between grazing and smoking (compared with no regular grazing, compulsive grazing OR = 1.59, 95% CI: 1.09, 2.33, *p* = 0.020; non-compulsive grazing OR = 1.04, 95% CI: 0.73, 1.49, *p* = 0.020); see Figure 1.

## 4. Discussion

To our knowledge, this study is the first to relate alcohol use, smoking, and ADHD symptoms to grazing, a potentially problematic and understudied eating pattern. Across alcohol use, smoking, and ADHD symptoms, there were consistent, significant positive associations with compulsive grazing; the association was strongest with ADHD symptoms, and with problematic alcohol use, although it was also present for lifetime smoking. The substantial association between compulsive grazing and ADHD symptoms is consistent with the growing body of literature on the significant comorbidity between ADHD and disordered eating. This finding represents a concerning issue, as the presence of ADHD can exacerbate eating pathology and complicate eating disorder treatment [48]. Conversely, those with ADHD have an increased prevalence of overweight and obesity, with dysregulated eating patterns being a likely contributor to weight gain [49]. This was corroborated in our study by the decrease in the strength of the association between positive ADHD screening and compulsive grazing with the adjustment for potential covariates, such as BMI. Compulsive grazing has been previously found to be associated with high BMI in both the Brazilian [6] and Australian [5] populations; hence, it may represent an eating pattern of particular concern in persons with ADHD.

As expected, impulsivity was independently associated with ADHD symptoms and compulsive grazing. When these were examined together, there was a substantial decrease in the strength of the association between compulsive grazing and ADHD symptoms when impulsivity was added to the model. This lends support to the hypothesis that impulsivity may be one of the mechanisms underlying both compulsive eating behaviours and ADHD. However, the relationship between compulsive grazing and ADHD symptoms, although diminished, remained significant, indicating that impulsivity was not the sole factor overlapping these constructs. Indeed, impulsivity was not found to mediate the relationship between ADHD symptoms and binge eating disorder symptoms in a non-clinical adult sample [48], indicating that other factors (for example, anxiety, depression, and emotion regulation) may also contribute to the significant relationship between ADHD and compulsive eating patterns.

A sizeable association was found between problematic alcohol use and compulsive grazing, which is consistent with existing research showing a significant overlap between substance use disorders and eating disorders; for example, studies have reported that 35% of persons with alcohol or illicit substance use problems have an eating disorder [50]. Furthermore, impulsivity was independently associated with problematic alcohol use. There was a small decrease in the strength of the association between compulsive grazing and problematic alcohol use when impulsivity was added to the model, indicating a smaller contribution of impulsivity to the relationship between this type of problematic eating and alcohol use and suggesting that other factors contribute to this relationship. For example, both alcohol use and grazing could be used for emotion regulation purposes. This finding is supported by the significant association between problematic alcohol intake and non-compulsive grazing independent of impulsivity, suggesting the possibility that the area of overlap may include an element of repetitive, prolonged intake (characteristic of non-compulsive grazing), which may present with reduced conscious awareness but without reaching compulsivity. 

There was a significant, albeit substantially smaller, association between compulsive grazing and lifetime smoking, which is also consistent with previous research noting a significant association between binge/purge-type eating disorders and higher rates of smoking and greater nicotine dependence [51]. In addition to its intrinsic reinforcing properties, nicotine—generally regarded as the major addictive psychoactive compound in tobacco)—is also particularly effective in establishing and increasing the salience of associated stimuli and in enhancing the reward value of other stimuli [52] such as palatable food; this process may drive the association with compulsive grazing. In addition, in those with eating disorders, tobacco is used as a method of weight control due to its appetite-suppressing properties and a distraction from food-related thoughts [50], which could also explain the link between lifetime smoking and grazing, although longitudinal research is needed to establish the temporal direction of these behaviours.

Unexpectedly, the association between lifetime smoking and impulsivity was non-significant, despite previously well-established connections between smoking and impulsivity [20]. Furthermore, the significant relationship between smoking and compulsive grazing did not change in magnitude when impulsivity was added to the model. The absence of a relationship in our study between impulsivity and smoking may reflect that only particular facets of impulsivity are implicated in tobacco use. For example, a recent meta-analysis using the UPPS-P model of impulsivity [53] found that impulsivity-related traits (such as reward sensitivity, lack of premeditation, and positive urgency) showed different patterns of association with smoking-related outcomes, such as smoking status and the severity of nicotine dependence [20]. It is possible that the single item used in this study to assess impulsivity did not cover all the different aspects of impulsivity or that the participants’ responses may have referred to different facets of this construct (i.e., some participants may have interpreted “impulsivity” as a lack of premeditation, while others may have interpreted it as a lack of perseverance, sensation seeking, etc.). Furthermore, it is possible that socioeconomic factors influenced this pattern of results. As in other countries, the use of tobacco products is concentrated in the poorest regions of Brazil [54], while Rio de Janeiro is one of the strongest economic areas. There is evidence that impulsivity has not only genetic but also environmental components related to economic circumstances and the availability of resources [55,56]; hence, it is possible that results from a lower socioeconomic area would have presented a different relationship between smoking and impulsivity. 

Our findings suggest that both grazing and substance use disorders may be underpinned by a predisposition to behavioural impulsivity or at least certain aspects of impulsivity. Compulsive grazing and increased substance use both contain an element of a perceived loss of control, which appears to be a transdiagnostic process in many problematic eating behaviours as well as substance use problems and disorders in which impulsivity is implicated. It is possible that the overlapping sense of loss of control reflects an underlying global cognitive difficulty with executive function skills, such as reduced inhibitory control. The association with symptoms of ADHD also suggests that other functions, such as inattention and delay aversion, may promote disordered eating behaviours such as grazing, possibly expressed through difficulties in adhering to a regular eating pattern.

Taken together, these findings highlight the need to assess for a comprehensive range of eating behaviours, including grazing, in those presenting with ADHD symptoms or problematic substance use and vice versa to consider the potential presence and impact of ADHD symptoms and substance use in those presenting with problematic eating patterns such as compulsive grazing. According to our findings and consistent with the existing literature [57], investigating several co-occurring conditions simultaneously instead of a single diagnosis is warranted. Shared vulnerabilities may be present for the development of co-occurring compulsive grazing and substance use difficulties, such as difficulties with executive function leading to increased impulsivity. Efforts should be made to identify those at risk for this comorbidity, given that the interplay between substance use and disordered eating contributes to higher psychopathology and treatment-related difficulties, such as symptom substitution, in which switching occurs from one problematic behaviour to the other [58]; longer recovery times; and higher rates of relapse [32]. It is also important to identify the predisposing factors for shared vulnerabilities, which may be neurobiological (such as differences in frontostriatal circuits or an inflammatory response to increased adiposity), social, or environmental (such as early-life adverse experiences).

This research presents several strengths, such as the large epidemiological sample that was representative of a major city in a middle-income country; the robust and multistage epidemiological design; and the use of validated psychometric measures to operationalise grazing, ADHD, and alcohol use. The limitations of this study include the use of a single item to measure impulsivity; the use of self-reported ADHD symptoms rather than clinical assessments to determine ADHD diagnoses; and a lack of information collection on genders other than male and female. The cross-sectional design also precludes conclusions regarding directionality and the temporal sequence of impulsivity and problematic eating or substance use. 

In conclusion, this study identified substantial associations between ADHD symptoms, problematic alcohol use and lifetime smoking, and compulsive grazing. Especially for symptoms of ADHD and alcohol use, there was an indication that impulsivity contributed to this association, suggesting a shared transdiagnostic vulnerability potentially related to executive function. These findings highlight the importance of assessing for compulsive grazing in those presenting with substance use or other conditions in which impulsivity plays a key role.

## Figures and Tables

**Table 1:** Sociodemographic and clinical characteristics.

Variables	*n*	%	95% CI ^5^
Gender			
Female	1407	51.8	49.0, 54.5
Male	890	48.2	45.5, 51.0
Race/ethnicity			
Mixed ^1^	987	43.2	40.3, 46.2
White	908	37.3	33.9, 40.9
Black	402	19.4	17.0, 22.1
Marital Status			
Single	860	35.9	32.5, 39.5
Married/Living as Married	1105	54.2	50.8, 57.6
Widowed/Divorced	332	9.8	8.5, 11.3
Education			
0–10 years	863	36.6	32.0, 41.4
11–14 years	990	46.3	42.7, 49.9
>14 years	444	17.1	14.2, 20.4
Employment status			
Student	108	6.8	5.3, 8.6
Paid employment	1450	63.5	60.2, 66.7
Not in paid employment	626	26.2	23.5, 29.2
Retired	109	3.4	2.5, 4.6
Income ^2^			
<BRL 1000	506	23.9	20.2,28.1
BRL 1001–3000	917	50.3	45.4, 55.2
>BRL 3000	479	25.8	21.6, 30.4
WHO BMI Category ^3^			
Underweight	62	3.7	2.7, 5.1
Normal Weight	685	31.7	28.6, 34.9
Overweight	824	36.5	33.7, 39.5
Obesity	609	28.1	25.2, 31.2
Grazing			
No regular grazing	1371	60.0	55.4, 64.4
Non-compulsive grazing	679	29.8	35.2, 44.3
Compulsive grazing	239	10.2	8.5, 12.3
Self-reported impulsivity	1048	42.9	38.1, 47.8
Problematic alcohol use	182	8.1	6.5, 10.1
Tobacco Use			
Never smoked	1663	75.0	72.8, 77.1
Current smoker	407	15.9	14.1, 17.8
Past smoker	227	9.1	7.7, 10.7
Positive ADHD Screening ^4^	101	4.5	3.7, 5.5

^1^ Mixed: Brown, Asian, and Indigenous, as defined in the Brazilian Demographic Census [47]. ^2^ BRL = Brazilian Real; BRL 1000.00 is the approximate minimum wage in Brazil. ^3^ BMI = body mass index, kg/m^2^ [40]. ^4^ ADHD = attention-deficit/hyperactivity disorder. ^5^ CI = confidence interval.

**Table 2:** Overlap between grazing and problematic alcohol use, lifetime smoking, impulsivity, and positive ADHD screening.

		No Regular Grazing *n* = 1371			Regular Non-Compulsive Grazing *n* = 679			Regular Compulsive Grazing *n* = 239			
		*n*	%	95% CI ^2^ (%)	*n*	%	95% CI ^2^ (%)	*n*	%	95% CI ^2^ (%)	*p*
Impulsivity	Yes	577	55.0	49.3, 60.6	316	29.7	25.0, 34.9	154	15.3	12.6, 18.4	<0.001
	No	794	63.7	58.6, 68.5	363	29.9	25.5, 34.6	85	6.4	4.8, 8.7	
ADHD ^1^ screening	Yes	31	30.7	21.7, 41.5	27	29.6	21.5, 39.3	43	39.7	30.9, 49.2	<0.001
	No	1340	61.3	56.7, 65.8	652	29.8	26.6, 33.9	196	8.9	7.1, 11.0	
Alcohol use problems	Yes	72	40.6	30.6, 51.3	72	40.6	31.8, 50.1	37	18.8	14.0, 24.8	<0.001
	No	1299	61.7	56.7, 66.4	607	28.8	24.9, 33.1	202	9.5	7.5, 11.9	
Lifetime smoking	Yes	355	56.8	49.5, 63.8	192	29.3	23.9, 35.3	85	14.0	11.0, 17.6	0.070
	No	1016	61.0	55.8, 66.0	487	30.0	25.7, 34.7	154	9.0	7.1, 11.3	

^1^ ADHD = attention-deficit/hyperactivity disorder. ^2^ CI = confidence interval.

**Table 3:** Crude and adjusted odds ratios and 95% confidence intervals from logistic regression analyses identifying associations between grazing and variables of interest.

	Non-Compulsive Grazing				Compulsive Grazing			
	Crude OR ^2^	95% CI ^3^	Adjusted OR	95% CI	Crude OR	95% CI	Adjusted OR	95% CI
Impulsivity								
Yes	1.15	0.88–1.51	1.06	0.79–1.42	2.75	1.89–4.00	2.01	1.40–2.87
No	1.00	-	1.00	-	1.00	-	1.00	-
ADHD ^1^ Screening								
Yes	1.98	1.15–3.43	2.45	1.41–4.26	8.94	5.11–15.63	5.66	2.53–12.69
No	1.00	-	1.00	-	1.00	-	1.00	-
Alcohol use problems								
Yes	2.14	1.29–3.56	2.13	1.23–3.70	3.02	1.65–5.53	3.33	1.72–6.43
No	1.00	-	1.00	-	1.00	-	1.00	-
Lifetime smoking								
Yes	1.05	0.73–1.50	1.19	0.78–1.81	1.67	1.12–2.47	2.02	1.33–3.07
No	1.00	-	1.00	-	1.00	-	1.00	-

^1^ ADHD = attention-deficit/hyperactivity disorder. ^2^ OR = odds ratio. ^3^ CI = confidence interval. Analyses were conducted with no regular grazing as the reference category. Adjusted ORs were calculated with potential covariates: gender, age, race, marital status, income, education, BMI, and presence of an eating disorder.

## Data Availability

The data presented in this study are available upon request from the corresponding author. The data are not publicly available, as this project is ongoing.
